# Differing effect of systemic anti psoriasis therapies on platelet physiology - a case report and review of literature

**DOI:** 10.1186/1471-5945-10-2

**Published:** 2010-03-31

**Authors:** Batya B Davidovici, Mary M Sullivan-Whalen, Patricia Gilleaudeau, James G Krueger

**Affiliations:** 1 Laboratory for Investigative Dermatology, The Rockefeller University, New York, NY, USA

## Abstract

**Background:**

Psoriasis is a common, chronic relapsing inflammatory skin disease. Lately, there is increasing evidence that psoriasis is more than "skin deep". Epidemiological studies showed that severe psoriasis might have also important systemic manifestations such as metabolic deregulations, cardiovascular disease (CVD) and increased mortality. Moreover, recently psoriasis patients were found to have platelet hyperactivity.

**Case Presentation:**

This is a case report and review of the literature. We present a patient with long standing severe psoriasis vulgaris with marked thrombocytosis. His thrombocytosis did not correlate with disease severity but rather with the different treatments that he was exposed to, subsiding only during treatment with anti Tumor Necrosis Factor (TNF)- agents. A literature review revealed that in rheumatoid arthritis, another systemic inflammatory disease; interleukin (IL)-6 might be implicated in causing thrombocytosis.

**Conclusion:**

This unique case report illustrates that different systemic treatments for psoriasis might have implications beyond the care of skin lesions. This insight is especially important in psoriasis patients in view of their deranged hemostatic balance toward a prothrombotic state, which might increase the risk of thrombosis and CVD. Therefore, further studies analyzing the effect of different drugs on platelets physiology are warranted.

## Background

Psoriasis is a chronic relapsing prevalent disease affecting 2-4% of the world's population [1-3]. Severe psoriasis is a disabling disease affecting the physical and emotional well-being of patients, and its effect on quality of life is similar to that seen with other major medical diseases such as diabetes, rheumatoid arthritis, and cancer [4,5].

Moreover, it is increasingly being recognized that in patients with severe or long-lasting disease, psoriasis is not merely a skin disease but is probably associated with other co-morbidities. Recently, studies based on large cohorts showed that severe psoriasis was association with metabolic derangements [6] and increased mortality [7]. Cardio-vascular diseases (CVD) were found to be the most common cause of death [8,9]. These data emphasize that the increased risk for occlusive vascular events in patients with severe psoriasis can be attributed not only to metabolic dysregulation, traditional lifestyle risk factors such as obesity, smoking or alcohol which have been also reported in these patients [10], but probably also to an independent risk directly resulting from the duration and severity of psoriatic inflammation.

We report a patient with long-standing severe psoriasis vulgaris with persistent reactive thrombocytosis that remitted only under anti TNF- therapy.

Recent data suggest that the hemostatic balance is deranged toward a prothrombotic state in psoriasis patients, which might be sustained by platelet hyperactivity, therefore this case pose a therapeutic dilemma concerning the need to treat reactive thrombocytosis in severe psoriasis patients.

## Case Presentation

In November 2006, a 36 years old male presented with severe plaque type psoriasis, to our hospital clinic. He has had psoriasis for the last 13 years; first it was localized to the scalp and periodically spread to other small areas of his body so he could control his disease with topical therapy. However, for the last six years the disease spread and covered the majority of his integument. He denied prior history of psoriatic arthritis. He had a positive family history of psoriasis, which affected his father, sister and paternal grandmother, but they all had a milder disease. His past treatments included various topical agents and several years ago he had a course of UVB with significant improvement, however he could not resume phototherapy because he lost his health insurance. Otherwise his past medical history was unremarkable. He had no known drug allergies and did not smoke. His current medications included over the counter iron pills. He denied any other systemic medications.

**Figure 1:**
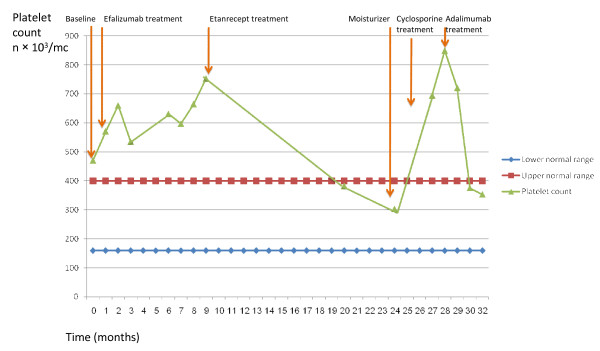
**Platelet counts during different anti psoriatic therapies in our patient**.

Examination revealed severely erythematous scaly, excoriated thick plaques involving approximately 80% of his body surface area, and pitting of his nails. The rest of his physical examination was unremarkable. After completion of the screening tests which were within normal limits except from mild normocytic anemia (HGB = 12.0 gr/d) and elevated platelets count (470 × 10^3^/mc, normal range 180-400 × 10^3^/mc) and receiving informed consent he was treated with efalizumab (Raptiva) 0.7 mg/kg SC once weekly and subsequently 1 mg/kg as per label. Under treatment his skin condition has gradually improved. However, after 3 months of treatment he had started to complain of arthralgia first involving his right ankle and latter on extending to 2 fingers in both hands and eventually to his left ankle. Treatment with Ibouprofen 600 mg twice daily did not relieve his pain. X-ray analysis confirmed the diagnosis of psoriatic arthritis therefore treatment with Efalizumab was interrupted after 9 months. In addition during the 9 months of efalizumab therapy his platelets count rose substantially (Figure 1). Then, etanercept (Enbrel) 50 mg twice weekly was started. After an initial flare of his skin condition, which was expected after cessation of efalizumab, a significant improvement of both his skin and joints ensued. Interestingly during the sixteen months of etanercept treatment his platelets count returned to normal. But unfortunately due to health insurance coverage problems he had to stop etanercept and consequently his skin conditioned dramatically deteriorated and resembled his baseline condition when he first reported to our clinic. Therefore cyclosporine (Neoral) 200 mg twice daily was introduced for approximately 2 months till he received his insurance approval for adalimumab (Humira) and again his skin and arthritis gradually improved. His platelets count rose while being off etanercept and even when he was treated with cyclosporine and his skin condition started to improve his platelets counts remained persistently high and only after adalimumab was started they returned to normal values.

## Results and Discussion

Thrombocytosis is classified as either primary (familial and clonal) or secondary. Unlike essential thrombocytosis, which is usually a form of myeloproliferative disease, reactive thrombocytosis is an exaggerated physiologic response to a primary stimulus thus it may accompany infections, iron deficiency anemia, hemorrhage, hyposplenism or chronic inflammatory diseases. Among other inflammatory diseases thrombocytosis was reported as a complication of inflammatory bowel diseases (IBD) [11,12] and rheumatoid arthritis (RA). However, in psoriasis, which is also a chronic inflammatory disease there is only one previous report of reactive thrombocytosis. That report included two patients with psoriasis who had episodes of fever, arthritis, and general fatigue [13]. Their symptoms seemed to be associated with increases in serum levels of platelet counts, IL-6, and elevated serum titers of C-reactive protein (CRP), which paralleled the severity of clinical symptoms. Platelet count has also been shown to correlate with disease activity in IBD. Similarly thrombocytosis usually occurs during the active clinical stages of RA and it was demonstrated to parallel disease activity [14].

Our case is unique since he had mildly elevated platelet counts at baseline, which continued to raise despite the improvement of his skin condition a result of efaluzimab treatment. This is specifically interesting since efalizumab, which is a humanized anti-CD11a monoclonal antibody (IgG1) that acts by blocking the interaction of lymphocyte function-associated antigen 1 with intracellular adhesion molecule 1, was described to cause an autoimmune thrombocytopenia in 0.3% of cases, based on data of the tolerance of 3,291 efaluzimab treated patients. There is only one other published case of a psoriasis patient that developed thrombocytosis while being treated with efalizumab [15]. However in that case the thrombocytosis diminished gradually, upon stopping the efalizumab, and after 9 weeks, returned to normal values. The onset mechanism of this phenomenon does not suggest an explanation. However, it is known that the activated platelets express CD11a [16]. In our patient, the thrombocytosis was probably not an adverse reaction to efaluzimab as it continued even after the drug was interrupted.

Efaluzimab is also known to infrequently cause new onset or recurrent severe arthritis events, including psoriatic arthritis. And indeed while on efaluzimab treatment our patient experienced severe arthritis, which was later on diagnosed to be psoriatic arthritis, and therefore this treatment was interrupted. The only case report of reactive thrombocytosis in psoriasis was in two patients who had also arthritis [13]. Therefore it is also possible that the development of psoriatic arthritis could have contributed to his rising platelet counts.

In our patient, the platelet count continued to rise while cyclosporine was administered with gradual improvement of his skin condition although, cyclosporine is reported to cause thrombocytopenia in 2% or less of the treated patients and there is no other case report of thrombocytosis during cyclosporine treatment.

In our patient platelet counts return to normal only during the periods that he was treated with anti TNFs (etanercept, adalimumab) and did not correlate with disease activity (Figure 1). The normalization of thrombocytosis was previously reported following treatment of RA patients with anti TNFs [17]. Attempts have been made to explain increments in the platelet counts. Some authors postulated that the symptoms and abnormal laboratory findings in these patients might be related to increased serum levels of IL-6, since IL-6 is a multi-potential cytokine with B-cell activating, T-cell activating, and thrombocytopoietic functions [13]. It was previously demonstrated that thrombocytosis secondary to the inflammation of RA can be modulated by proinflammatory megakaryocyte-related interleukins, IL-6, IL-1b and IL-4 [18,19]. In patients with active inflammatory bowel disease, Heits et al. [20], disclosed that the levels of thrombopoietin (TPO), which is the main regulator hormone of platelet production, were significantly increased in association with thrombocytosis and elevated IL-6 levels. Furthermore, it was demonstrated that IL-6-induced thrombocytosis in mice is accompanied by enhanced hepatic TPO mRNA expression and elevated TPO plasma levels [20]. In addition administration of IL-6 to cancer patients resulted in a corresponding increase in TPO levels [20]. Similarly others have also identified positive correlations between platelet count and IL-6 and between TPO and IL-6 [21]. Yet in their study they confirmed that TPO acts as an acute phase protein but excluded the possibility that it is uniquely responsible for thrombocytosis of inflammatory disorders. This might recognize in IL-6 a credible candidate as a cooperating factor.

Interestingly, in a relatively big study of 219 patients with psoriasis and psoriatic arthritis, IL-6 was significantly higher in patients with psoriasis and inflammatory joint disease compared with patients having psoriasis of the skin [22].

It should be remembered that Il-6 has also a central role in the pathogenesis of psoriasis as T memory/effector cells (Tmem/eff) isolated from psoriatic patients are chronically activated and poorly suppressed by regulatory T cells (Treg). IL-6, which signals through Stat3, allows escape of Tmem/eff cells from Treg-mediated suppression in a murine system. In a recent study, Goodman et al [23], showed that IL-6 is elevated and most highly expressed by CD31(+) endothelial cells and CD11c(+) dermal dendritic cells (DCs) in lesional psoriatic skin. They found that IL-6, but not other Stat3-activating cytokines, was necessary and sufficient to reverse human T cell suppression by Treg in an in vitro model using activated DCs as a source of IL-6. They identified cells within lesional tissue that co-express CD3, IL-17, and IL-6, indicating that Th17 cells are present in vivo within the psoriatic Tmem/eff population and contribute to IL-6-mediated resistance to Treg suppression.

Cyclosporine is a calcineurin inhibitor that was reported to increase IL-6 production and IL-6 mRNA expression [24]. In fact this is suspected to be the mechanism by which cyclosporine induces renal damage and that the use of an IL-6-neutralizing antibody may be useful in reducing cyclosporine-induced renal damage [25]. Moreover cyclosporine-upregulated IL-6 gene expression in vivo, may explain in part the molecular mechanisms responsible for cyclosporine -induced gingival overgrowth [26]. In a previous study cyclosporine induced IL-1β expression in circulatory leukocytes and this might be sufficient to induce IL-6 production in some tissues [27]. This is also probably the reason of the sustained and even increase in platelet counts seen in our patient during cyclosporine treatment.

Anti TNF- however down-regulates the production of IL-1, IL-6, IL-8, and GM-CSF [28,29]. The reductions in circulating IL-6 in anti TNF-treated RA patients were reaching significance from the first day of treatment [30]. Therefore anti TNFs might normalize platelet counts through the reduction of circulating IL-6 levels.

Furthermore, increased platelet activation and aggregation have been demonstrated to be the features of IBD and has been proposed to contribute to the pathogenesis of mucosal inflammation seen in the condition [31]. In psoriasis patients, evidence for an in vivo platelet activation, which could contribute to the development of thrombotic events, has also been established [32]. Spontaneous platelet hyperaggregability, mean platelet volume, plasma levels of β-thromboglobulin and platelet factor 4, which are markers of platelet activation, were found to be significantly higher in psoriasis patients compared with that in controls. Interestingly, these markers, as well as platelet aggregability were significantly reduced after psoriasis had cleared [33]. Moreover, platelet regeneration time, measured as malondialdehyde (MDA) recovery after aspirin ingestion, was significantly shorter in psoriasis patients [34,35]. Finally, P-selectin expression by platelets was also increased in psoriasis patients, showing a direct correlation with disease severity [36]. Simultaneously, activated platelets may play a role in psoriasis pathogenesis by favoring leukocyte rolling in the skin microvasculature [36] and platelet derived 12- hydroxyeicosatetraenoic acid may increase keratinocyte DNA synthesis [37].

Finally, there is a therapeutic dilemma because reactive thrombocytosis (even at this level) is often not treated, as the chance of thrombosis is very rare. However, it is important to bear in mind that severe psoriasis itself is a risk factor for metabolic derangements [6], cardiovascular diseases and thrombosis [7-9] probably as a result of the chronic inflammatory nature of the condition. Thus, some hematologists tend to give an anti-platelet agent or low molecular weight heparin in these circumstances especially if there is another coexisting risk factor for thrombosis [38]. Until more is known about the patho-physiology of the increased risk for CVD in psoriasis patients, such a therapeutic approach might be recommended.

## Conclusion

Different anti psoriasis therapies might have different effects on platelet physiology. In view of the increased risk of thrombosis and CVD in severe psoriasis patients further studies analyzing the effect of different drugs on platelets physiology are warranted.

## Competing interests

The authors declare that they have no competing interests.

## Authors' contributions

All persons who meet authorship criteria are listed as authors:

BBD participated in the design, analysis and wrote the manuscript. MMSW and PG helped with the acquisition of data and manuscript revision with the acquisition of data and manuscript revision. JGK participated in the design, analysis and revised the manuscript. All authors have given final approval of the version to be published

## Pre-publication history

The pre-publication history for this paper can be accessed here:

http://www.biomedcentral.com/1471-5945/10/2/prepub
